# Clustering Acoustic Segments Using Multi-Stage Agglomerative Hierarchical Clustering

**DOI:** 10.1371/journal.pone.0141756

**Published:** 2015-10-30

**Authors:** Lerato Lerato, Thomas Niesler

**Affiliations:** Department of Electrical and Electronic Engineering, University of Stellenbosch, Western Cape, South Africa; Institute of Psychology, Chinese Academy of Sciences, CHINA

## Abstract

Agglomerative hierarchical clustering becomes infeasible when applied to large datasets due to its *O*(*N* ^2^) storage requirements. We present a multi-stage agglomerative hierarchical clustering (MAHC) approach aimed at large datasets of speech segments. The algorithm is based on an iterative divide-and-conquer strategy. The data is first split into independent subsets, each of which is clustered separately. Thus reduces the storage required for sequential implementations, and allows concurrent computation on parallel computing hardware. The resultant clusters are merged and subsequently re-divided into subsets, which are passed to the following iteration. We show that MAHC can match and even surpass the performance of the exact implementation when applied to datasets of speech segments.

## Introduction

Clustering can be described as the process of finding the natural grouping(s) of a set of patterns or objects based on their similarity [1]. This can be achieved through the use of clustering algorithms which are broadly classified into two groups: *hierarchical* and *partitional* [2–4]. Partitional clustering methods seek to divide the data without considering how the final clusters may themselves be combined into larger groups, or be subdivided into smaller groups. They are based on the optimisation of an appropriate objective function that quantifies how well the clusters represent their members [1]. The most common example of a partitional method is the k-means algorithm. For partitional approaches, the number of clusters must be known beforehand, and this can present major challenges [5]. When we have no prior knowledge of the number of clusters, hierarchical methods are a favourable choice [6, 7]. In contrast to partitional approaches, these methods consider how clusters can be subdivided into sub-clusters or be grouped into super-clusters, thereby providing a hierarchical assignment of objects into groups. Among hierarchical methods, one can further distinguish between divisive and agglomerative approaches. The former are based on a succession of data splits that continues until each data object occupies its own cluster [8, 9]. Agglomerative hierarchical clustering (AHC) on the other hand, is a bottom-up approach that initially treats each data object as a singleton cluster and successively merges pairs of clusters until a single group remains [4, 10]. This process begins by computing a pairwise similarity between all *N* points in the dataset, leading to a symmetric *N* × *N* similarity matrix. Since the entries on the main diagonal are zero, only upper or lower triangular matrix entries need to be computed. A major disadvantage of the AHC algorithm is the *O*(*N* ^2^) space complexity [5, 11] associated with the storage of this triangular distance matrix.

We will focus on the agglomerative hierarchical clustering of acoustic speech segments such as phonemes. These segments are time series of varying length and are not easily represented as vectors in a vector space. However, they can be compared and similarities can be computed. Since AHC requires only these similarities and not a vector representation, it is well suited to our application. Even for fairly small speech databases, however, the number of phonemes can run into hundreds of thousands. While there is no strict threshold above which one can declare the amount of data as *large*, there is no doubt that the direct application of AHC is intractable in terms of memory for this application [5, 12].

Clustering of phonemes, triphones or any other form of acoustic segments has found useful application in the field of automatic speech recognition (ASR) [13–16]. Our over-arching focus is the development of automatic speech recognition systems for under-resourced languages [17]. In particular, we aim to automatically discover phoneme-like acoustic units from raw acoustic speech data. This is useful for languages for which a phoneme inventory may not yet have been defined, and labelled data may not be available. In the long term, we aim to develop ASR based on automatically-determined acoustic units instead of the traditional reliance on phonetic knowledge.

We propose a multi-stage AHC (MAHC) method that splits the AHC problem into sub-problems which can be executed independently. This reduces the space complexity when the sub-problems are executed sequentially and allows faster computation when parallel computing hardware is available. The algorithm requires only the pairwise distances between object to be known, and hence makes the clustering of substantial speech databases feasible. The algorithm does not require the number of clusters to be specified in advance, and is shown to be comparable in performance to parallel spectral clustering (PSC) [18] which has also been developed specifically for the processing of large datasets.

## Related Work

The problem of clustering large data using agglomerative hierarchical techniques has been addressed by other researchers in various ways. We note the early work by Narasimha and Krishna [19] which proposes a multilevel technique for clustering datasets. While their approach is in some respects similar to ours, it has been tested only on a fairly small dataset consisting of 50 manually generated samples in a 2-dimensional Euclidean space. This data is split into *P*_1_ sub-groups. AHC is applied to each sub-group; in each case yielding *C* clusters (a value of *C* = 5 was used in all experiments). After this ‘first level’, a representative cluster from each of the 5 *P*_1_ sub-groups is determined and stored as a ‘data point’ for level two. Subsequently, level-two data is further divided into *P*_2_ sub-groups, and AHC is applied to each. This procedure continues until the predetermined number of levels, *K*, has been reached, and it is shown that the optimal value of *K* can be mathematically determined. An alternative way of automatically finding *K* for the same method is reported by Suresh [20]. Here the authors show that standard AHC is computationally more expensive than the proposed technique based on the two level process.

Tang *et al* [21] also propose a distributed hierarchical clustering algorithm for large data. Their aim is to improve and minimise the storage requirements of traditional hierarchical algorithms for parallel computing architecture. They use a threshold on the similarity determined by a human expert to classify data items as unrelated, thereby making the similarity matrix sparse. The sparse similarity matrix is used to sequentially create disjoint sets of closely related data items. Each disjoint set is clustered in parallel, forming its own sub-clusters. Finally a single linkage method is used to measure similarity between these sub-clusters, which are subsequently themselves subjected to AHC to complete the final dendrogram. The technique is tested on the MPC Orbit (MPCORB) dataset which contains approximately 380,000 asteroids, each of which is represented by a 6-dimensional feature vector. The major variable here is the threshold on the similarity and the authors show experimentally how it affects the number of disjoint sets and also the execution time at each step of the algorithm.

Cobo *et al* [22] employ a subspace clustering paradigm [23] which assumes that high dimensional data objects lie around a union of subspaces such that clustering can be performed independently in each subspace. The data used in this work represent the activities performed by learners in an online discussion forums. A total of 3842 written posts were captured from 672 students over a period 333 days. Each student is represented by a multi-dimensional feature vector which represents attributes relating to their participation in online discussions. The features are classified as coming from either a reading or writing domain. The feature vector attributes relating to the writing domain include: the ratio of reply posts written by a learner relative to the total number of reply posts, and the ratio of learners who replied at least once relative to the total number of learners. The feature vector attributes relating to the reading domain include: the ratio of posts read by a learner as a fraction of total number of posts and the ratio of threads where a learner reads at least one post relative to the total number of threads. These data are clustered using AHC in a ‘first stage’ where learners with similar activity patterns are grouped together separately in each domain. The AHC acquires normalised Euclidean distance pairs as input and calculates inter-cluster similarities using the complete linkage method. The second stage of the method entails the grouping together of those learners who belong to the same clusters in both reading and writing. The participation profiles of the learners are mapped to the final clusters by observing and comparing the values of the parameters that characterise the learners’ activity patterns in each cluster. The participation profiles are known before hand. This work was subsequently advanced by Cobo [24] by including more domains.

The second tier of literature relevant to our work emanates from research that focuses specifically on the clustering of acoustic speech segments. Paliwal [14] considers the clustering of acoustic segments by first representing each segment with its centroid and then applying the k-means algorithm to these centroids. A codebook containing *N* entries where each entry defines one acoustic sub-word unit (acoustic segment) cluster is generated. A similar process is reported by Lee *et al* in [25].

Wang *et al* [16] convert data into segment-level Gaussian posteriograms (SGP’s). These SGP’s are obtained from the segment likelihoods generated by a Gaussian mixture model which has been trained on all the training data. Each acoustic segment is represented as a sequence of posterior probabilities of Gaussian components given the observation sequence. Data is then consolidated in a distance matrix of size *M* Gaussians by *N* segments. The authors separately cluster both the segments and the Gaussian components of the matrix using the *normalized cut* [26] approach with a known number of clusters. A confusion matrix of acoustic segment labels is used for evaluation together with the F-measure and the mormalised mutual information [27]. Although the method described by these authors does not employ AHC, it does target the same application we have chosen.

One clustering approach that has been extended specifically to partition large datasets is spectral clustering (SC) [28, 29]. A typical spectral clustering algorithm acquires pairwise distances from *N* *d*-dimensional data points located in a Euclidean space, ${\mathbb{R}}^{N}$, and constructs a dense similarity matrix $Y\in {\mathbb{R}}^{N\times N}$. In some cases *Y* can be modified to be a sparse matrix. Subsequently, the Laplacian matrix, *L* = *Z* − *Y*, is computed where *Z* is a diagonal matrix whose entries are row/column sums of *Y*. SC requires the number of clusters, *K*, to be specified so that the first *K* eigenvectors of *L* can be computed and stored as columns of a new matrix, $V\in {\mathbb{R}}^{N\times K}$. Finally the k-means algorithm is used to cluster the *N* rows of the matrix *V* into *K* groups. SC as described is not suitable for application to large datasets [29] because of the storage requirement associated with the *N* × *N* similarity matrix. Chen *et al* [18] propose an extension to spectral clustering that makes the processing of large datasets possible. In the proposed parallel spectral clustering (PSC) algorithm, data is split into *N*/*p* subsets, where *p* is the number of compute nodes. The datasets used by the authors to evaluate PSC comprise 193,844 instances of document data (RCV1) and 2,121,863 instances of photo data (PicasaWeb). The number of nodes *p* is varied from 1 to 64 for RCV1 and from 16 to 256 for PicasaWeb. On each processor, the similarity matrix *Y* for local data is computed and the *t*-nearest neighbours are determined from each row/column of *Y*. The matrix *Y* is then sparsified and made symmetric. Two ways of approximating Y are proposed: the sparsifying criterion and the Nyström approximation. The Nyström approximation method computes eigenvectors of a *b* × *b* (*b* ≪ *N*) sub-matrix of *Y* with the goal of using these eigenvectors to approximate the eigenvectors of *Y*. Chen *et al* found that the sparsification approach yields better results than the Nyström approximation. The resulting eigenvector matrix, *V*, is stored on distributed nodes. Experiments were performed on the Google distributed clusters using the MapReduce parallelisation system.

## Agglomerative Hierarchical Clustering

### Data representation

In the cluster analysis literature, a dataset, **X**, is generally depicted as a composition of *N* objects that must be partitioned into *K* clusters. Object data are generally represented in the form $\text{X}=\{{\text{x}}_{1},{\text{x}}_{2},{\text{x}}_{3},...,{\text{x}}_{N}\},{\text{x}}_{i}\in {\mathbb{R}}^{d}$, where each data point is represented by a *d*-dimensional feature vector such that the complete dataset is viewed in the form of an *N* × *d* *pattern matrix* [2, 3, 30]. Similar data points are clustered according to a similarity function *D*(**x**_*i*_, **x**_*j*_) which has the following properties:

1. *D*(**x**_*i*_, **x**_*i*_) = 0, ∀*i*
2. *D*(**x**_*i*_, **x**_*j*_) = *D*(**x**_*j*_, **x**_*i*_), ∀*i*, *j* (*i* ≠ *j*)
3. *D*(**x**_*i*_, **x**_*j*_) ≥ 0.

The input to the AHC algorithm is a similarity matrix populated by the values of *D*. The properties of *D* listed above lead to a lower or upper triangular similarity matrix with *M* = *N*(*N* − 1)/2 independent entries below or above the leading diagonal.

The above data representation does however not accommodate time series data objects. A time series data object consists of a set of multidimensional Euclidean points, each corresponding to a successive instant in time. We therefore present a more generic dataset in Eq 1.

$$\begin{array}{c} X=\{X_{1},X_{2},X_{3},...,X_{N}\} \end{array}$$ (1)

Here **X**_*i*_ = {**x**_*i*1_, **x**_*i*2_, **x**_*i*3_, …, **x**_*in*_} is a time series object and *n* is the number of consecutive Euclidean points in the object **X**_*i*_. When objects are simply Euclidean points, we would have the special case **X**_*i*_ = {**x**_*i*1_}. This representation reflects a more general dataset for objects in ${\mathbb{R}}^{d}$. Under this formulation the similarity between two objects is therefore given by *D*(**X**_*i*_, **X**_*j*_).

### Clustering algorithm

We formulate our problem as that of *N* data objects to be grouped into *K* partitions using an AHC algorithm [2]. Let the set of clusters be C, such that $C=\{C_{1},C_{2},\ldots ,C_{K}\}$ where **C**_*k*_ is a subset whose membership ideally comprises similar objects. In addition, **C**_*p*_∩**C**_*q*_ = ∅ for *p*, *q* = 1, 2, …, *K* where *p* ≠ *q*, while $C_{1}\cup C_{2}\cup \ldots \cup C_{K}=X$ as set out in Eq 1. The symbols ∩, ∪ and ∅ indicate set intersection, set union and the empty set respectively.

**Fig 1:**
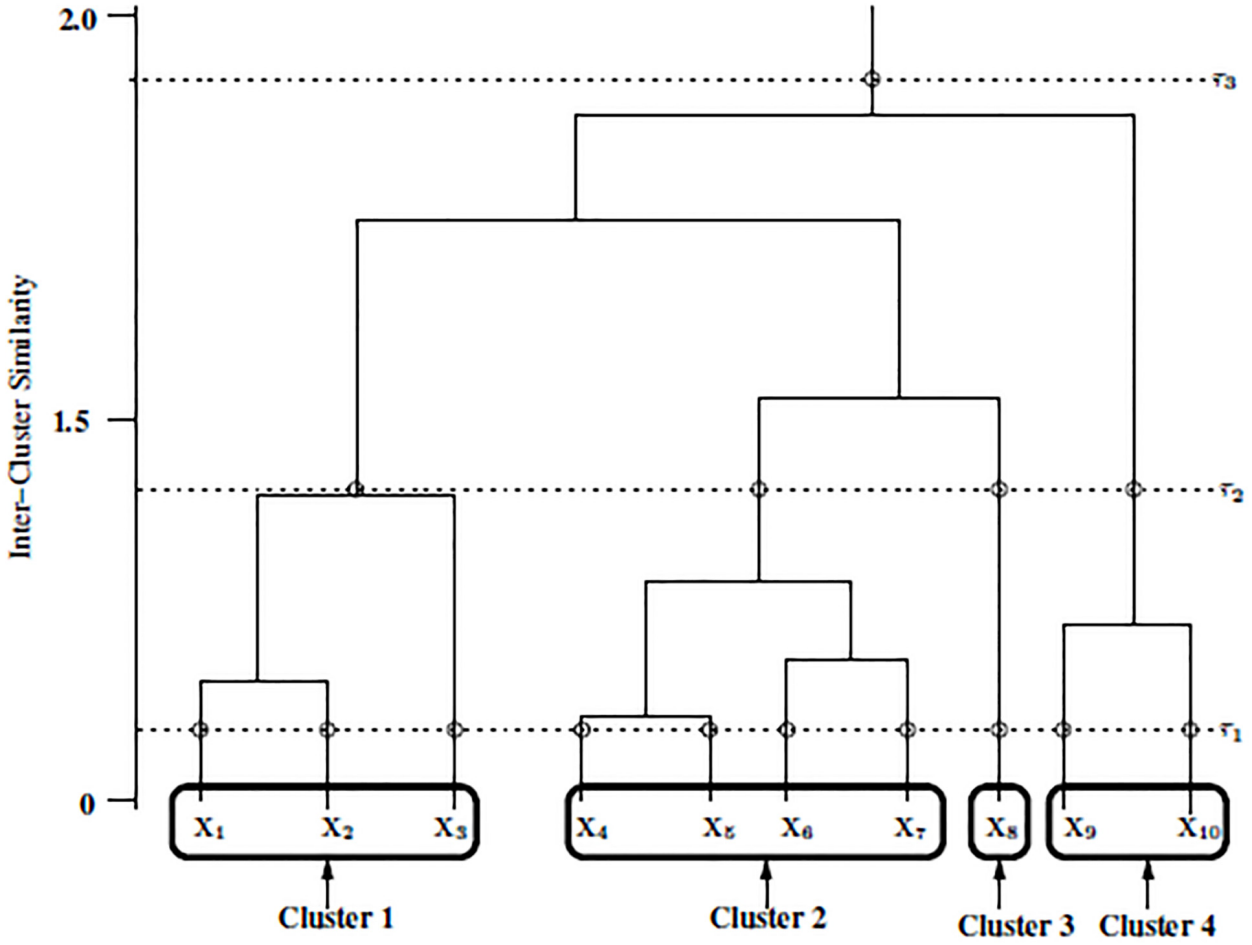
An example of a dendrogram.

In AHC, the agglomeration of data objects is initialised by the assumption that each object is the sole occupant of its own cluster. Starting from this initial single-occupancy scenario, a binary tree structure referred to as a *dendrogram* is created by successively merging the closest cluster pairs until a single cluster remains [6]. Illustrated in Fig 1, the dendrogram is a structure consisting of many *U*-shaped lines that connect data points into a hierarchical tree.

The height of each *U* represents the similarity or distance between the two clusters being merged. To cluster the data, the dendrogram is *cut*, by placing a threshold on this similarity. In Fig 1, three possible thresholds τ_1_, τ_2_ and τ_3_ are shown. This threshold is often referred to as the *cutoff* of the dendrogram.

Inter-cluster distances are normally calculated using linkage methods or the sum of squares [3]. Among linkage methods, single, average and complete linkages are common choices. We have chosen the Ward method [31, 32] to calculate inter-cluster similarity, both because it has been found by other researchers to outperform other linkage methods [2] and because we have found it to lead to consistently better results during initial experimentation. The Ward method is a minimum variance criterion that minimises the total within-cluster variance. It utilises the squared distance between cluster centres or the Euclidean distance between the individual objects.

A generic formulation that incorporates several merging criteria used in AHC is given by the *Lance-Williams dissimilarity update* formula [6, 7]:

$$\begin{array}{c} \begin{array}{c} D\left(\left(C_{i}\cup C_{j}\right),C_{k}\right)=\alpha_{i}D\left(C_{i},C_{k}\right)+\alpha_{j}D\left(C_{j},C_{k}\right)+\beta D\left(C_{i},C_{j}\right)\\ +\gamma |D\left(C_{i},C_{k}\right)-D\left(C_{j},C_{k}\right)| \end{array} \end{array}$$ (2)

where $\alpha_{i}=\frac{N_{i}+N_{k}}{N_{i}+N_{j}+N_{k}}$, $\alpha_{j}=\frac{N_{j}+N_{k}}{N_{i}+N_{j}+N_{k}}$, $\beta =-\frac{N_{k}}{N_{i}+N_{j}+N_{k}}$ and *γ* = 0.5 define the agglomerative criterion using the Ward method. *N*_*i*_, *N*_*j*_ and *N*_*k*_ denote the number of objects in cluster **C**_*i*_, **C**_*j*_ and **C**_*k*_ respectively. It has been found that the Ward method exhibits computational and space complexity of *O*(*N* ^2^) [32, 33].

## Multi-stage Agglomerative Clustering

The AHC algorithm described in the previous section requires the computation and storage of *M* = *N*(*N* − 1)/2 similarities. For large *N*, this becomes impractical. In the following we present an approximation to the AHC algorithm that we refer to as multi-stage agglomerative hierarchical clustering (MAHC) as a way of addressing this practical limitation. Our three main objectives are:

1. to design and test a parallelisable hierarchical agglomerative clustering (AHC) strategy, called MAHC, on a manageable-sized dataset.
2. to compare the results of the MAHC with the classical AHC method.
3. to evaluate both the AHC and MAHC scenarios using both internal and external methods [34] namely the L method [35] and the F-measure [36] respectively.

**Fig 2:**
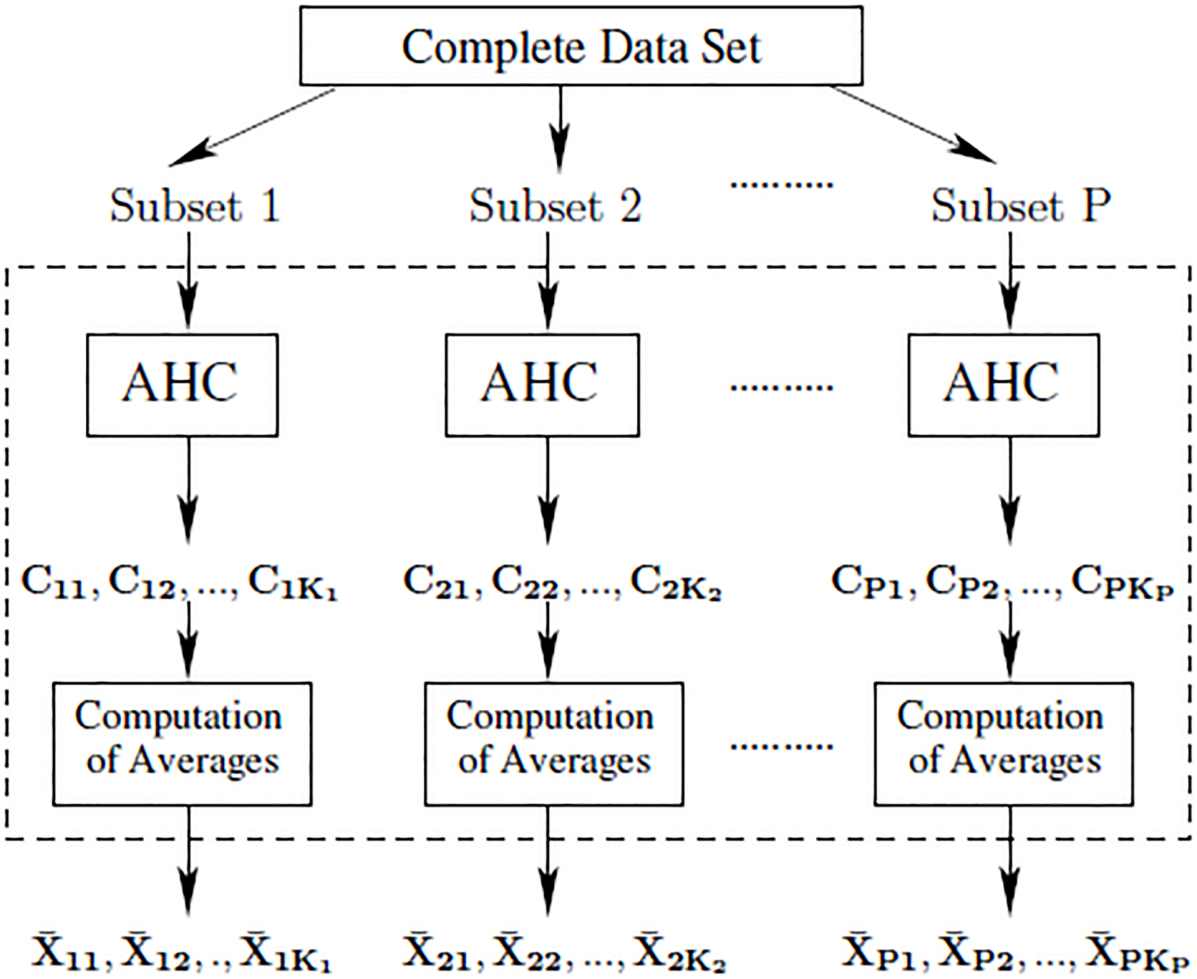
The first stage of MAHC algorithm.

Our proposed method is a two stage iterative process. The first stage divides the complete dataset into *P* subsets and applies AHC to each subset. These *P* clustering operations can occur sequentially or concurrently in parallel. Fig 2 illustrates the first stage.

The value of *P* is heuristically determined by assessing the available memory of the computational resources based on the size of the dataset. In the first stage, each subset of data is processed as follows:

1. AHC is applied to subset *p* and generates a set, $C_{p}$, of *K*_p_ clusters where *p* = 1, 2, …, *P* and $C_{p}=\{C_{p1},C_{p2},\ldots ,C_{pK_{p}}\}$.
2. An average point, ${\bar{\text{X}}}_{p}$, is determined for each cluster, where ${\bar{\text{X}}}_{p}=\{{\bar{\text{X}}}_{p1},{\bar{\text{X}}}_{p2},...,{\bar{\text{X}}}_{pK_{p}}\}$.

The average, ${\bar{\text{X}}}_{p}$, can be a mean, median, mode, medoid or any other measure of locality which represents all objects in a cluster. From the first stage architecture we can approximate the computational complexity as *P* × *O*(*N* ^2^/*P* ^2^). This is an improvement by a factor *P* over the standard AHC algorithm whose complexity is *O*(*N* ^2^).

The second stage clusters the averages. At the beginning of the second stage, the total number of average objects that must be clustered is *S* = *K*_1_ + *K*_2_ + … + *K*_*P*_. We denote the set of all data in the second stage as $\bar{X}=\{{\bar{\text{X}}}_{1},{\bar{\text{X}}}_{2},...,{\bar{\text{X}}}_{S}\}$. We then apply AHC to $\bar{X}$ and determine *K* clusters of the averages. The purpose of this step is to merge similar clusters resulting from the first stage. Since the data were divided randomly into subsets at the top of Fig 2, the *P* separate clustering processes performed in parallel may result in some clusters that are similar.

**Fig 3:**
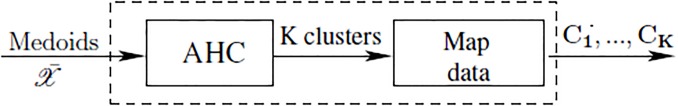
The second stage of MAHC algorithm.

All data elements from the first stage are mapped to their corresponding averages, to obtain the final object clusters, **C**_1_, **C**_2_, …, **C**_*K*_. The second stage process is illustrated in Fig 3.

**Fig 4:**
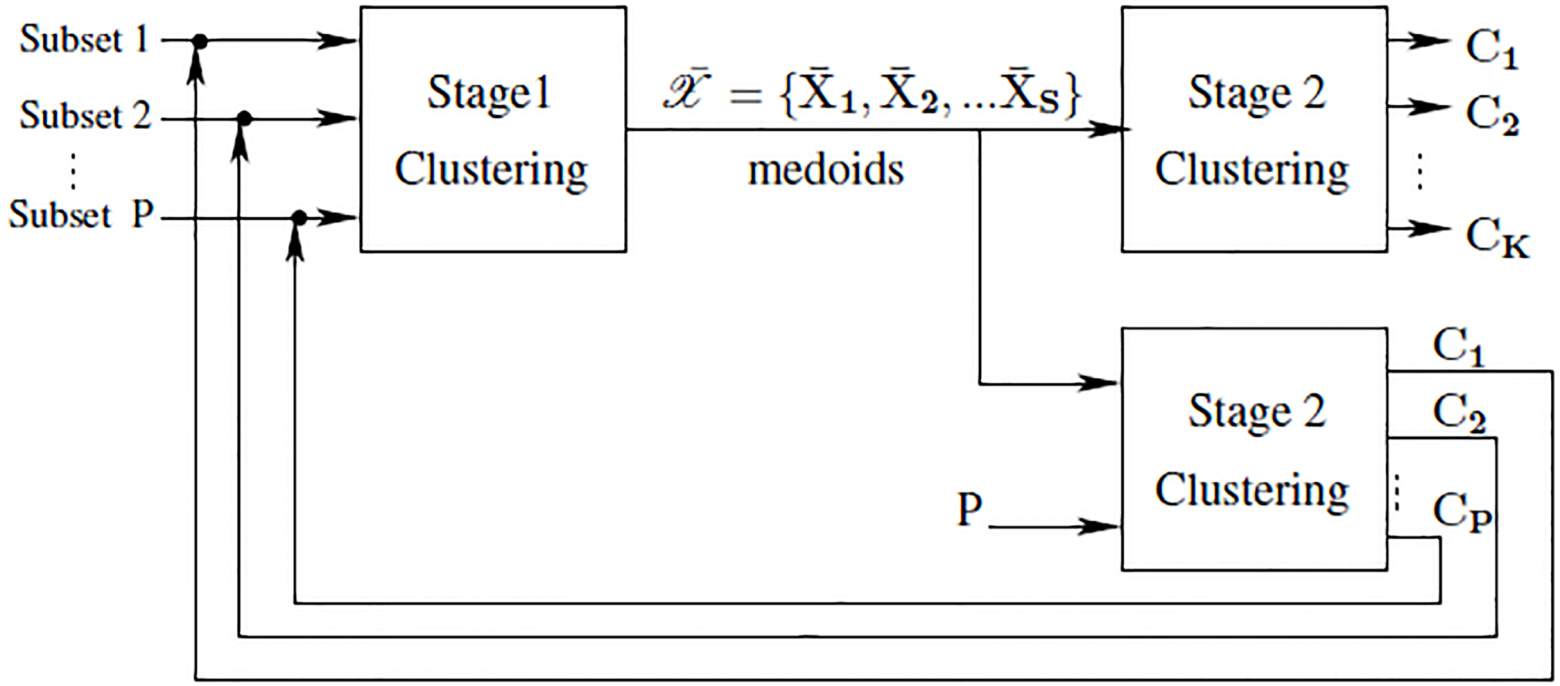
The complete MAHC algorithm.

The final step in our MAHC algorithm is the regeneration of the *P* subsets at the top of Fig 2, thereby rendering the MAHC algorithm iterative. This is done by setting *P* = *K* and mapping the data to each of the *new* *P* subsets according to the clusters **C**_1_, **C**_2_, …, **C**_*K*_ obtained after the first iteration of stages 1 and 2. The motivation for this iteration is that, by grouping similar clusters in stage 2 and using those to redefine the subsets from which stage 1 proceeds, each independent clustering operation constituting stage 1 will process data that are more self-similar and that are different from the data processed by the other *P* clustering operations. If this succeeds, the division into independent clustering operations in stage 1 becomes an increasingly appropriate strategy. During the last iteration of MAHC, stage 2 produces the final *K* clusters. The complete process is shown in Fig 4.

The parameters of the proposed MAHC algorithm are:

1. The number of subsets, *P*
2. The number of clusters each subset is divided into during stage 1, *K*_i_
3. The final number of clusters, *K*
4. The number of iterations of the MAHC algorithm.

The effect of these parameters will be investigated experimentally in the following sections. In particular, it will be shown that the number of clusters *K* can be estimated automatically.

## Acoustic Segment Clustering

### Acoustic segments as data items

Acoustic segments are temporally bounded intervals of speech data that correspond to potentially meaningful sound classes, such as phonemes or sequences thereof [37]. They are vector time series of variable length representing a short period of the speech audio signal. We aim to partition *N* such acoustic segments (objects) into *K* clusters. We will assume that each segment is represented by a sequence of feature vectors, as commonly used to represent speech for automatic speech recognition. Let **X**_i_ denote the *i*-th acoustic segment such that **X**_*i*_ = {**x**_1_, **x**_2_, **x**_3_, …, **x**_*n*_*i*__} and ${\text{x}}_{t}\in {\mathbb{R}}^{d}$ where *t* = 1, 2, …, *n*_*i*_. The pattern matrix of the *i*-th acoustic segment is therefore of size *d* × *n*_*i*_. The collection of all segments constitutes the acoustic database X as depicted in Eq 1.

In this case the similarity function, *D*(**X**_*i*_, **X**_*j*_) computes the distance between two pattern matrices **X**_i_ and **X**_j_ with equal number of rows (*d*) but generally different number of columns (*n*_i_ and *n*_j_). When comparing this formulation of the distance with that of Euclidean data objects, it becomes apparent why AHC is a natural choice of clustering algorithm for the case of speech segments. Since the segments, **X**_i_, are matrices of variable dimension, they are not easily represented in a fixed-dimensional space. Hence it is difficult to visualise these data, and algorithms requiring the computation of centroids, like k-means, are hard to apply. However, AHC requires only that the distances between data points are known. To measure these distances, we use the dynamic time warping (DTW) algorithm, which is a popular similarity measure used in speech processing [38, 39]. DTW recursively determines the best alignment between the two segments by minimizing a cummulative cost that is commonly based on the Euclidean distances between time aligned time-series vectors. Other distances such as Manhattan and Mahalanobis can also be employed. Based on preliminary experimentation, we selected Manhattan distance as a similarity measure between feature vectors. Eq 3 shows how the DTW distance between two segments **X**_i_ and **X**_j_ is computed.

$$\begin{array}{c} \delta \left(\psi ,\omega \right)=∥X_{i}\left(\psi \right),X_{j}\left(\omega \right)∥+\min\left\{\delta \left(\psi ,\omega -1\right),\delta \left(\psi -1,\omega -1\right),\delta \left(\psi -1,\omega \right)\right\} \end{array}$$ (3)

In Eq 3, *δ*(*ψ*, *ω*) is the minimum accumulated distance along any path from (1, 1) to (*ψ*, *ω*), *ψ* = 1, …, *n*_*i*_, *ω* = 1, …, *n*_*j*_ and *i*, *j* are segment indices. The score of the best alignment *δ*(*ψ*, *ω*) serves as a similarity measure between **X**_i_ and **X**_j_.

### Clustering acoustic segments with MAHC

Acoustic segments are divided into *P* subsets and a similarity matrix for each subset is calculated using the DTW algorithm. The first stage of MAHC is applied to the subsets as described previously. The average depicted in Fig 2 in this case is a medoid. A medoid is the cluster member, ${\bar{\text{X}}}_{p}$, which is, on average, closest to all other members, and is computed as follows:

$$\begin{array}{c} {\bar{X}}_{p}=\underset{p,p\neq q}{arg min}\sum_{q=1}^{K_{p}}D\left(X_{p},X_{q}\right) \end{array}$$ (4)

We use medoids as a representation of each cluster because we have no information on the shape of the cluster and we cannot determine cluster centroids. We consider the DTW distance between the medoids of two clusters to be a measure of inter-cluster similarity that can be used as input similarity matrix for AHC in the second stage. Finally all acoustic segments are mapped to their corresponding medoids to obtain the final set of clusters.

## Evaluation Mechanisms

We will evaluate the performance of the MAHC approach by applying it to the TIMIT speech corpus, for which a phonetically labelled segmentation is provided. Since this ground truth is available, external evaluation metrics [40] can be used to evaluate the quality of the clusters. External metrics use the prior knowledge about the data; usually in the form of labels, to asses the quality of the experimentally determined clusterings [27]. However, since our aim is to extend the work to speech datasets for which such labels are not available, we will also characterise performance using internal metrics [34]. These are based only on the information intrinsic to clustered data and do not require ground truth labels.

We first consider the cardinality *K*_i_ of each sub-process at the first stage of the MAHC algorithm shown in Fig 2. We chose the F-measure [36] for external and the L Method [35] for internal validation respectively. The F-measure is widely used for the evaluation of clustering and classification systems [41]. The L method was selected for internal validation because it yielded reasonable results in our experimental evaluation, it is computationally cheap, and it has received considerable attention by the research community [42].

The F-measure scores provide a benchmark against which the L method can be measured because we have not yet been in a position to test the clusters found by the MAHC algorithm in a speech recognition system.

### The F-Measure

The F-measure assumes that each acoustic segment, **X**_i_, has a known label (commonly termed the class) representing the ground truth [3, 36, 41]. We will use this measure to quantify the quality of a division of the acoustic segments in the dataset into one of *K* clusters. The F-Measure is based on the measures recall and precision for each cluster with respect to each class in the dataset.

Assume that for class *l* and cluster *k* we know *n_kl_*, the number of objects of class *l* that are in cluster *k*; and *n_k_* is the total number of objects in cluster *k* and *n_l_* is the number of objects in class *l*. The precision and recall are given by Eqs 5 and 6 respectively.

$$\begin{array}{c} Precision\left(k,l\right)=\frac{n_{kl}}{n_{k}} \end{array}$$ (5)

$$\begin{array}{c} Recall\left(k,l\right)=\frac{n_{kl}}{n_{l}} \end{array}$$ (6)

Precision indicates the degree to which a cluster is dominated by a particular class, while recall indicates the degree to which a particular class is concentrated in a specific cluster. The F-measure, *F*, is calculated as follows:

$$\begin{array}{c} F\left(k,l\right)=\frac{2\times Recall(k,l)\times Precision(k,l)}{Recall(k,l)+Precision(k,l)} \end{array}$$ (7)

where *k* = 1, 2, …, *K* and *l* = 1, 2, …, *L*. An F-measure of unity indicates that each class occurs exclusively in exactly one cluster; a perfect clustering result.

In preliminary experiments, we used the F-measure to determine an appropriate cutoff for each dendrogram during the first stage. We however do not use the F-measure for experiments involving large data because its calculation is computationally demanding. This is due to the need to compute the F-measure for a large number of possible clusters for each dendrogram. For example, if there are *N* = 100 acoustic segments, the F-measure must be computed for up to 99 possible cutoff values which corresponds to the number of possible clusterings. For each clustering the F-measure involves *K* × *L* iterations within which it searches inside each cluster for objects of class, *l*. This exhibits *O*(*N* ^2^) complexity and makes the F-measure slow as the number of data points increases.

### The L Method

**Fig 5:**
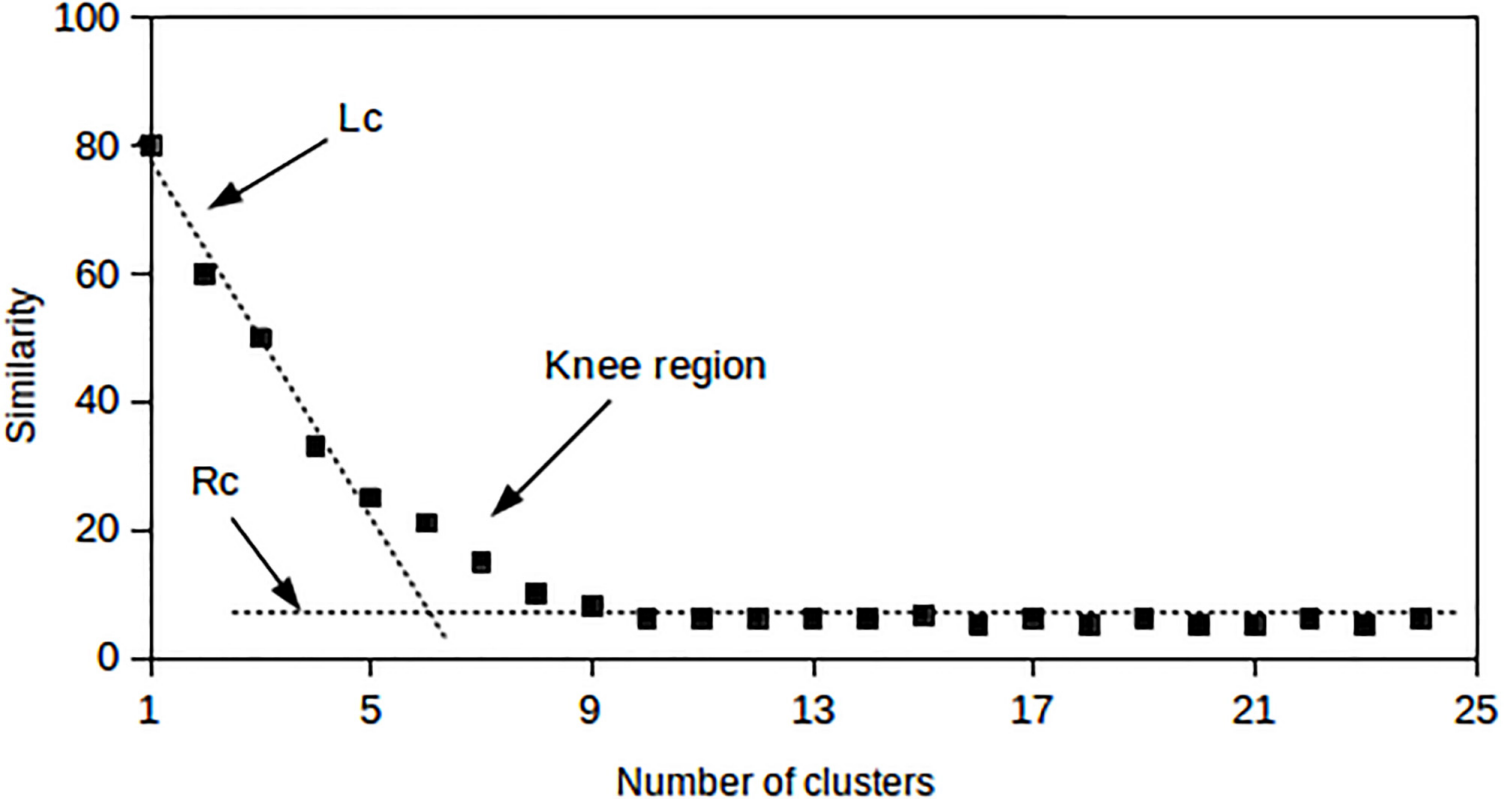
Best-fit lines to locate the knee of the graph in the L method.

Cluster similarity measures in a dendrogram such as the Ward method produce a knee-shaped graph when plotted against the number of clusters as illustrated in Fig 5.

It has been hypothesised that the optimum number of clusters occurs at the *knee* of this plot [34]. Hence the location of the *knee* can be used to estimate the optimal number of clusters even when no ground truth is available. Fig 5 demonstrates one method by means of which the location of the knee may be determined.

In our investigation we use the L method implementation proposed by Salvador and Chan [35] which estimates the number of clusters at the knee region. This implementation separates regions of the curve into two parts, namely *Lc* and *Rc*. These are left (*Lc*) and right (*Rc*) sequences of data points partitioned at a point where *x* = *c* and *x* represents a number along the x-axis. *Lc* ranges from *x* = 2 to *x* = *c*. A point at *x* = 1 is normally ignored because one cluster is not a useful result. *Rc* includes points with *x* = *c* + 1, …, *b*, where *c* = 3, …, *b* − 2. To estimate the optimal knee point, the root mean square error at *c* (*RMSE*_c_) is used such that the partition of *Lc* and *Rc* is at *x* = *c*. *RMSE*_c_ is calculated as shown in Eq 8:

$$\begin{array}{c} RMSE_{c}=\frac{c-1}{b-1}\times RMSE\left(Lc\right)+\frac{b-c}{b-1}\times RMSE\left(Rc\right) \end{array}$$ (8)

where *RMSE(Lc)* is the root mean square error of the best-fit line on the left of the knee and *RMSE(Rc)* the same to the right of the knee. The lines *Lc* and *Rc* shown in Fig 5 intersect where the value of *c* minimises the *RMSE*_c_. The value of *c* at that point is considered to be at the optimal number of clusters. Since *R* can have a very long tail, some authors suggest that the data is truncated [35].

### Determining a threshold for the dendrogram

**Fig 6:**
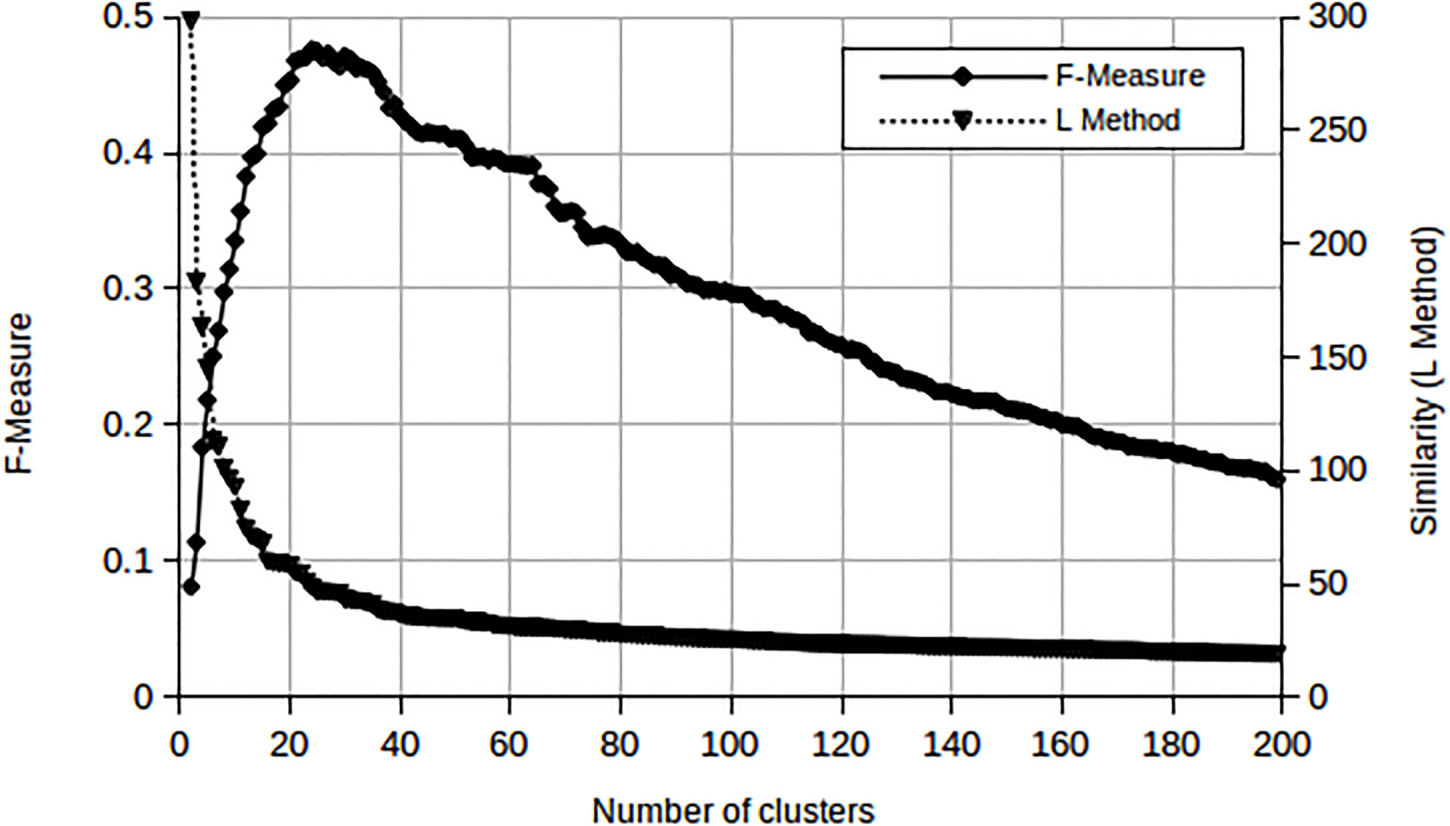
AHC results of a small experiment with 29 true clusters. The peak in the F-measure occurs at 24 clusters, while the knee of the L Method is found at 22 clusters.

One way to determine the best cutoff for a dendrogram is to calculate the F-measure at all possible threshold values and then determine the number of clusters at the peak. Alternatively, the number of clusters can be estimated by locating the knee of the similarity measure graph (L Method). Both are shown in Fig 6, which is a result of a small experiment in which 754 acoustic segments were clustered using the classical AHC method. The true number of classes in this case is 29 and the F-measure peak occurs at 24 clusters.

This experiment demonstrates that the F-measure increases with the number of clusters, reaches a peak, and then begins to decline as the number of clusters increases. This eventual decline is due to the rise in single occupancy clusters. When applying the L method to the same data, the knee was located at 22 clusters. We observe that both the F-measure and the L method produce a comparable number of clusters.

## Experiments, Results and Discussion

### Data

All experiments used acoustic segments taken from the popular and well-established TIMIT speech corpus [37, 43]. TIMIT contains a total of 6300 sentences recorded from 630 speakers. Each speaker reads 10 sentences, the first 2 of which are identical across all speakers in the database. To avoid bias, these two sentences have been excluded in all our experiments. The TIMIT corpus is chosen because it includes time-aligned phonetic transcriptions meaning that both phonetic labels and their start/end times are provided. We will consider triphones [37], which are phones in specific left and right contexts, as our desired clusters. We used a maximum of 42 base phones in our experiments.

From the TIMIT data we have compiled 4 datasets, varying in size. Table 1 shows the number of segments (objects) in each dataset, as well as the true number of classes, the range of class cardinality and the total number of feature vectors.

**Table 1:** Composition of experimental data. *N* indicates the total number of segments, *L* the total number of classes (unique number of triphones), *R* the frequency of occurrence of each triphone, *V* the total number of feature vectors in ${\mathbb{R}}^{39}$ and *M* = *N*(*N* − 1)/2 is the number of similarities which must be computed for straightforward application of AHC.

**Dataset**	**Segments (N)**	**Classes (L)**	**Range (R)**	**Points (V)**	**Entries (M)**
Small Set A	17 611	280	50–373	274 677	0.16 × 10^9^
Small Set B	17 640	636	26–49	301 026	0.16 × 10^9^
Medium Set	54 787	1 387	20–373	910 189	1.5 × 10^9^
Large Set	123 182	19 223	1–373	2 193 793	7.6 × 10^9^

**Fig 7:**
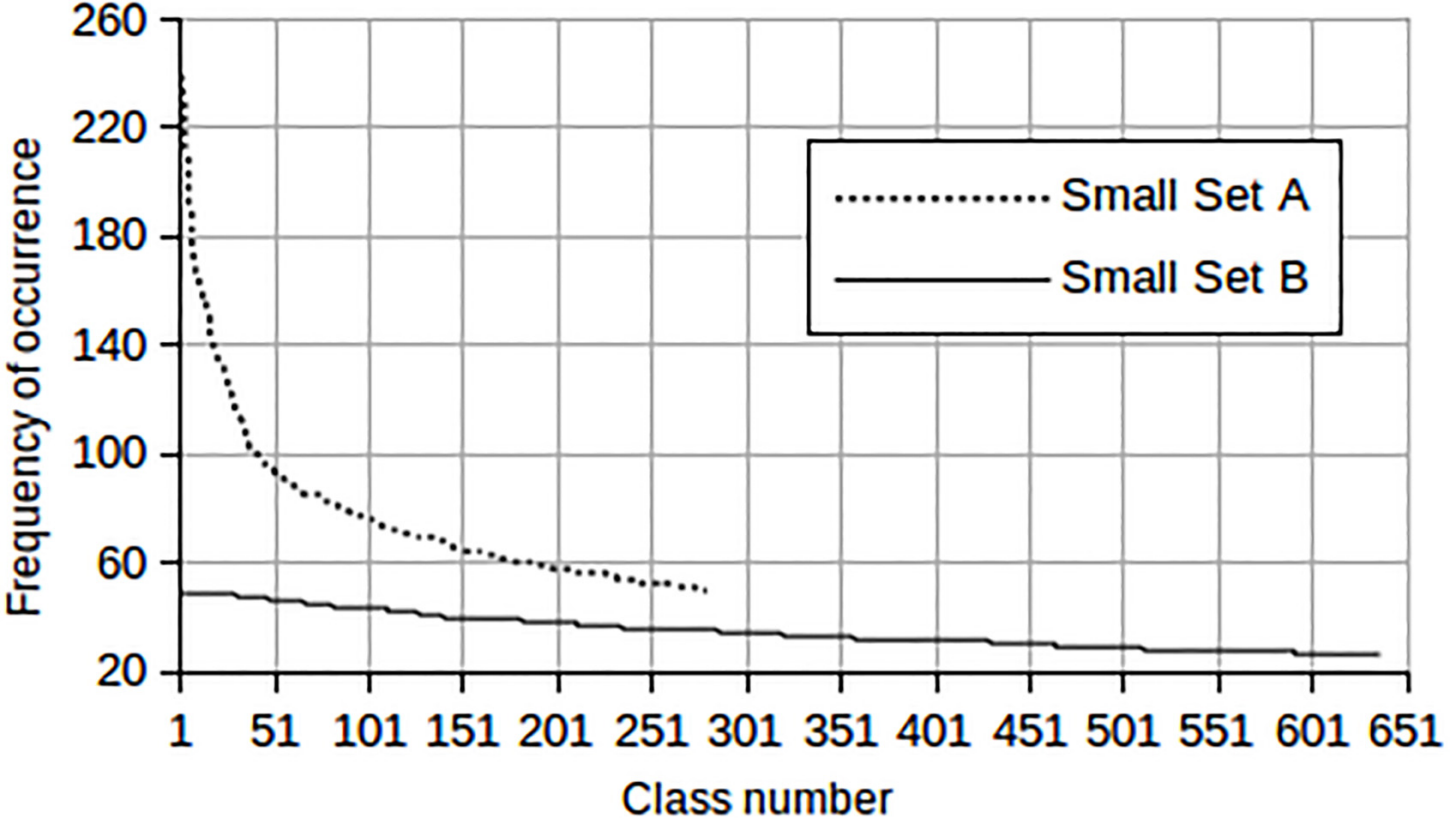
Distribution of the number of segments per class for the two independent Set A and Set B.

Small Set A and Small Set B differ in their class distribution as depicted in Fig 7. Small Set A is more skewed compared to Small Set B. This means that in Small Set A, some classes have many more members than others. The class membership range is shown in Table 1. The Medium Set and the Large Set are skewed in the same fashion as the Small Set A, since this is the type of distribution one may expect in unconstrained speech.

During data preparation, each acoustic segment is represented as a series of 39 dimensional feature vectors consisting of 12 Mel frequency cepstral coefficients (MFCCs), log frame energy, and their first and second differentials. The MFCC’s were chosen on the basis of their well-established popularity in high performance speech processing systems [12]. Feature vectors are extracted from data frames that are 10ms in length, and consecutive frames overlap by 5ms (50%). The MFCC’s were computed using a HTK [44].

### AHC Baseline

Baseline results were obtained by applying classical AHC to the small and medium datasets described in Table 1. In each case the dendrogram was cut so as to optimise the F-measure as shown in Table 2. Subsequently the L method was applied to determine the dendrogram thresholds, and these results are reflected in Table 3. Even when the number of clusters is obtained using the L method, we can still apply the F-measure to asses the final result for comparison. In the case of the large dataset, the excessive size of the similarity matrix did not allow the application of classical AHC. For this case, results will only be shown for the MAHC method.

**Table 2:** Baseline results when the cutoff is determined via the F-measure.

**Dataset**	**Optimal no. of clusters**	**AHC: F-measure**	**PSC: F-measure**
Small Set A	144	0.1104	0.1198
Small Set B	577	0.0662	0.0655
Medium Set	717	0.0476	0.0265

**Table 3:** Baseline results when the cutoff is determined via the L method and the output is evaluated with the F-measure.

**Dataset**	**Optimal no. of clusters**	**AHC: F-measure**	**PSC: F-measure**
Small Set A	162	0.1074	0.1290
Small Set B	163	0.0529	0.0546
Medium Set	503	0.0456	0.0317

### Parallel spectral clustering benchmark

In order to benchmark our results, we have also applied parallel spectral clustering (PSC) as proposed by Chen *et al* [18] to our datasets. This algorithm can also be applied in situations in which only the similarities between objects are known. Furthermore, in contrast to other variants of spectral clustering, PSC can be applied to large datasets. PSC does however require the number of clusters *K* to be specified. In benchmark comparisons we will therefore always employ the number of clusters used in the corresponding MAHC experiment. We employ *t* = 20 nearest neighbours for small and medium sets, and *t* = 100 for the Large Set, as suggested by the experiments in [18]. Additionally we also show how parallel spectral clustering (PSC) performs at the baseline conditions in Tables 2 and 3. We observe that PSC delivers better performance than classical AHC for Small Set A, while the two approaches exhibit similar performances for Small Set B. AHC offers improved performance on the Medium Set.

### Small datasets

Since the clustering experiments are computationally demanding, we begin experimentation with the small sets (A and B). Subsequently we extend our investigation to the larger datasets. The first experiments applied classical AHC, to the 17,611 segments of Small Set A and to the 17,640 segments of Small Set B. Subsequently, we split these datasets into 2, 4 and 6 subsets and in each case performed 10 iterations of the MAHC algorithm. In each case the number of clusters was chosen by maximising the F-measure both after stage 1 and stage 2. This number of clusters was also used as input to parallel spectral clustering (PSC) to provide a benchmark.

**Fig 8:**
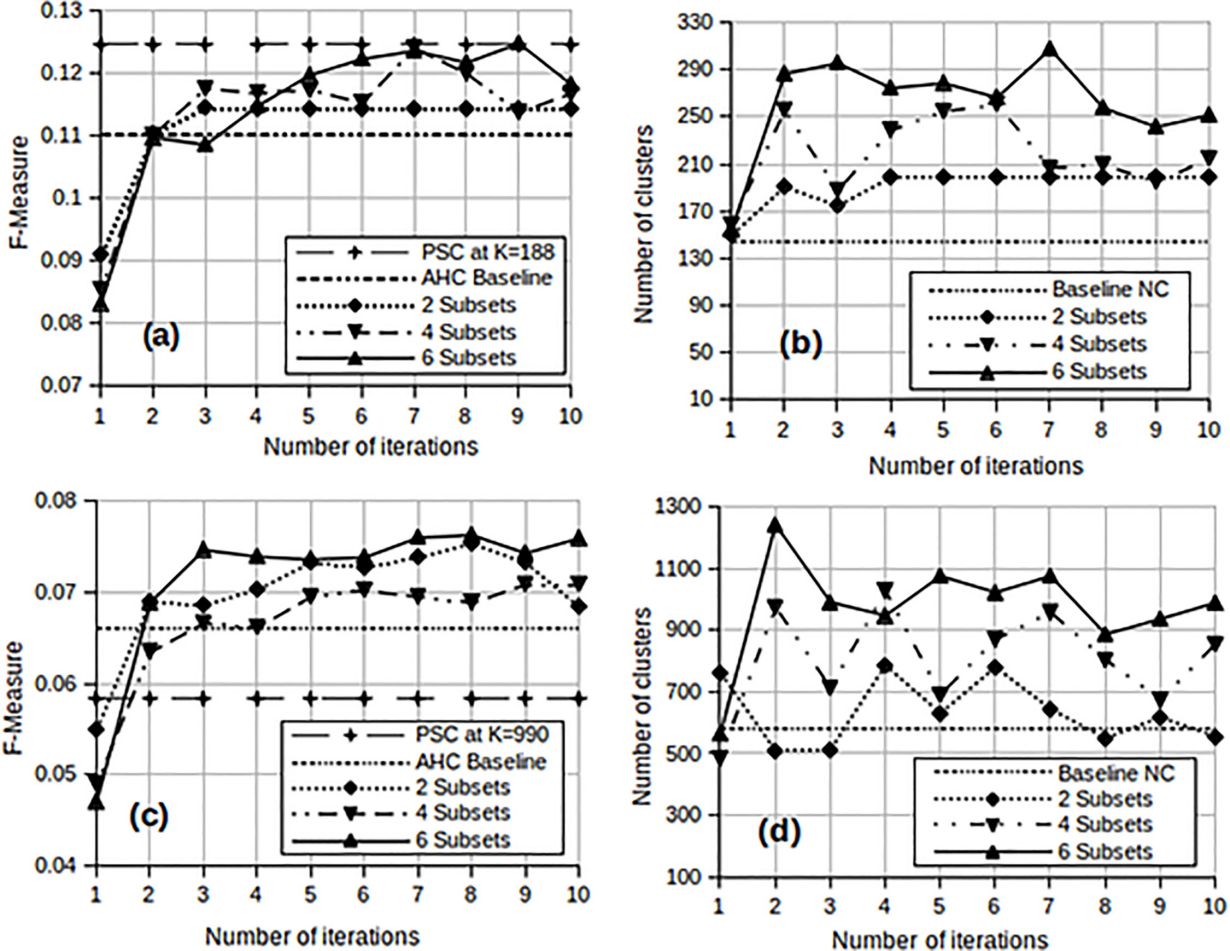
Performance of MAHC and PSC for the small sets in terms of F-measure, using F-measure to determine thresholds in stage 1. (a) F-measure for Small Set A (b) MAHC optimal number of clusters for Small Set A (c) F-measure for Small Set B (d) MAHC optimal number of clusters for Small Set B.

The results are shown in Fig 8 for each successive iteration both in terms of F-measure and the optimal number of clusters.

Fig 8(a) shows that, for Small Set A, the F-measure generally increases with each iteration, and that it exceeds the performance achieved by the AHC baseline at the third iteration. Fig 8(b) shows the number of clusters produced after each iteration of the MAHC algorithm. As mentioned earlier, these clusters are obtained by optimising the F-measure both at stage 1 and at stage 2. As a baseline, the number of clusters obtained when optimising the F-measure for the classical AHC method is also shown (see Table 2). Fig 8(b) shows that the number of clusters obtained by the MAHC algorithm is larger than that obtained by the AHC method, and that it varies somewhat from iteration to iteration. The performance of PSC at *K* = 188 in terms of F-measure is also shown in Fig 8(a) and generally outperforms MAHC. Details on how the value of *K* was chosen are discussed in later sections.

The same trends are observed for Small Set B in Fig 8(c) and 8(d). However, Fig 8(d) shows that the number of clusters determined by the MAHC method fluctuates more widely. This may be owing to the number of classes relative to the distribution of Small Set B data. Despite this fluctuation, the quality of the clusters, in terms of F-measure, are consistently above the baseline from the third iteration onwards. Furthermore, the performance of PSC with *K* = 990 in Fig 8(c) for Small Set B is surpassed by MAHC from the second iteration onwards.

**Fig 9:**
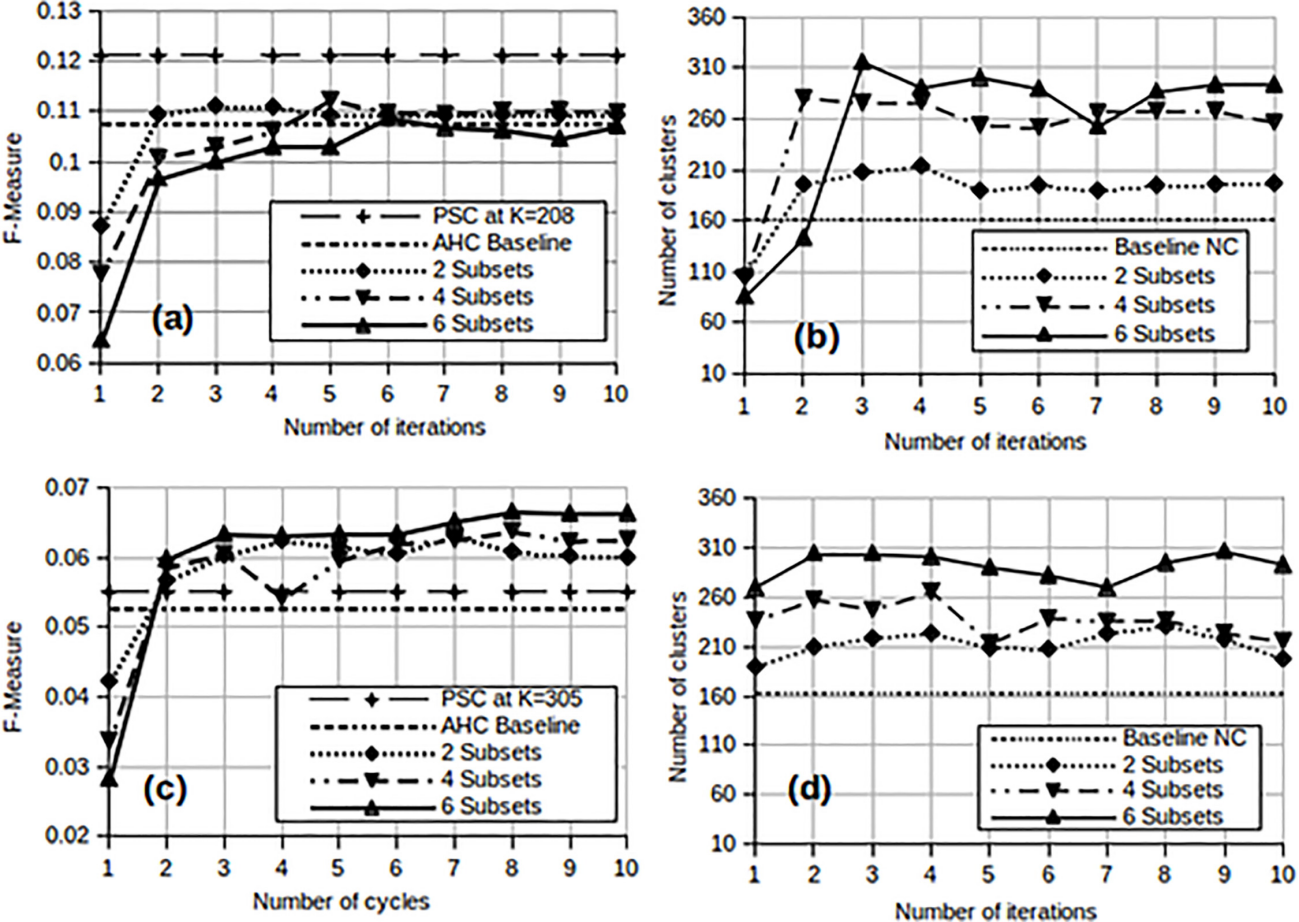
Performance of MAHC for the small sets in terms of F-measure, using the L method to determine thresholds in stage 1. (a) MAHC and PSC F-measure for Small Set A (b) MAHC optimal number of clusters for Small Set A (c) MAHC and PSC F-Measure for Small Set B (d) MAHC optimal number of clusters for Small Set B.

The experiments shown in Fig 8 were repeated, this time using the L Method to determine the threshold for the dendrogram in stage 1. Thresholds in stage 2 continued to be chosen by optimising the F-measure. The results are presented in Fig 9.

From Fig 9(a) we observe that the MAHC surpasses the baseline AHC for Small Set A at the fourth iteration except in the case of 6 subsets. The F-measure achieved by PSC for Small Set A at *K* = 208 in Fig 9(a) remains the best overall. Fig 9(c) mirrors the performance trends observed in Fig 8(c) for Small Set B in which the MAHC performance is equal to or better than the AHC baseline from the second or third iteration. Furthermore, MAHC improves the performance of PSC after the first iteration. Fig 9(b) and 9(d) also indicate a much more stable number of clusters than we have observed in Fig 8. These results continue to show that the number of clusters generally increases with the number of subsets. This may again be due to the the distribution of the data. In general we observe from both Figs 8 and 9 that, with small datasets, the MAHC matches or even surpasses the performance of AHC after 3 or 4 iterations. As shown in Fig 8, MAHC is able to improve on PSC in one experiment (Small Set B), but not in the other (Small Set A).

### Medium dataset

**Fig 10:**
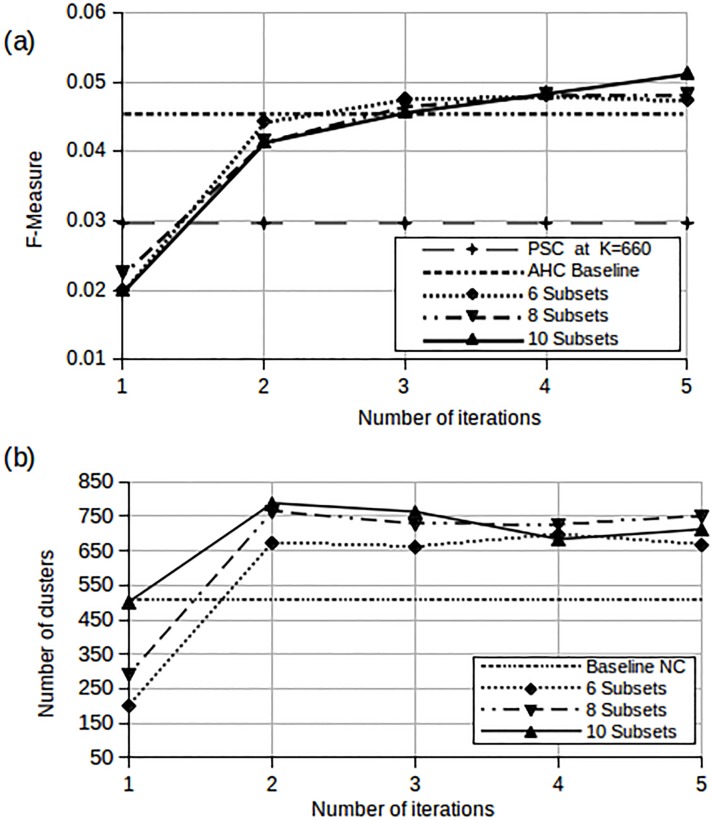
Performances for the Medium Set. (a) MAHC and PSC F-measure (b) MAHC optimal number of clusters (NC).

The Medium Set, which is approximately three times larger than the small sets, is still small enough for classical AHC to be applied. We used this set to verify and support our findings with the small datasets. The L method was used to determine the dendrogram cutoff in stage 1 for computational reasons. However we continued to use the F-measure in stage 2 of the MAHC as way of objectively evaluating cluster quality. We also show the performance of parallel spectral clustering (PSC) for the Medium Set at the same value of *K*. A set of experiments, similar to those reported in Fig 9, were performed for the medium set and the results are displayed in Fig 10.

These results show that the performance of the MAHC method closely approximates that of the AHC baseline from the third iteration onwards. This is consistent with our findings for the two smaller sets. At the third iteration, MAHC produces 660 clusters, and we therefore use this value for the PSC benchmark. For the Medium Set, we observe in Fig 10(a) that MAHC improves on the performance of PSC in terms of the F-measure.

Fig 10(b) shows that, as observed with Small Set A and Small Set B, the number of clusters (NC) produced by the MAHC exceeds that produced by classical AHC. The latter finds *K* = 503 while the former suggests between 660 and 786 clusters after 2 iterations. However, in contrast to the smaller sets, the number of clusters determined by MAHC is fairly stable. This may be due to the higher cluster occupancy which is in turn due to the larger volume of data.

Another important observation made after the experiments with the small and medium sets is that the number of clusters produced by stage 1 coincides closely with the number of clusters in stage 2 after a second iteration. Using the notation introduced in Fig 2, we can express this observation as:

$$\begin{array}{c} \sum_{i=1}^{P}K_{i}\approx K \end{array}$$ (9)

where *K* is the number of clusters (NC) produced by MAHC. Part of this observation is substantiated in Tables 4, 5 and 6.

**Table 4:** Relation between experimental number of clusters (*K*) and the sum of NC’s from each subset of Small Set A using the L method.

	Number of clusters (NC) per subset in each iteration				
Subset(i)	Iteration 1	Iteration 2	Iteration 3	Iteration 4	Iteration 5
1	46	81	67	85	113
2	47	82	62	57	48
3	54	40	59	54	30
4	44	14	32	25	40
5	53	49	71	34	26
6	50	56	25	36	44
Σ**(K_i_)**	294	322	316	291	301
**K**	87	144	316	291	301

**Table 5:** Relation between experimental number of clusters (*K*) and the sum of NC’s from each subset of Small Set B using the L method.

	Number of clusters (NC) per subset in each iteration				
Subset(i)	Iteration 1	Iteration 2	Iteration 3	Iteration 4	Iteration 5
1	44	45	91	55	61
2	51	37	36	35	52
3	51	67	27	67	36
4	39	75	49	57	69
5	48	50	48	58	44
6	41	33	54	30	29
Σ**(K_i_)**	274	305	305	302	291
**K**	271	305	305	302	291

**Table 6:** Relation between experimental number of clusters (*K*) and the sum of NC’s from each subset of Medium Set using the L method.

	Number of clusters (NC) per subset per iteration				
Subset(i)	Iteration 1	Iteration 2	Iteration 3	Iteration 4	Iteration 5
1	110	189	87	171	134
2	104	144	165	142	137
3	96	121	131	137	84
4	104	48	98	88	131
5	100	99	105	79	83
6	108	71	74	79	97
Σ**(K_i_)**	622	672	660	696	666
**K**	200	672	660	696	666

Here we have verified that the relation in Eq 9 holds for all the results shown in Figs 8, 9 and 10. Although this observation should be thoroughly investigated in further experiments, it allows the number of clusters at each level of the MAHC algorithm to be chosen in an unsupervised manner; using the L Method in stage 1 and Eq 9 in stage 2. Our benchmark PSC results are consequently obtained using this observation to determine the required value of *K*.

### Large dataset

From Table 1, we see that the application of classical AHC to the large dataset would require the computation and storage of 7.6 × 10^9^ similarities. This is infeasible both from a storage and computational point of view on most current computing hardware. We apply the MAHC algorithm to this dataset, splitting it into 10 subsets. As before, the L method is used to determine the number of clusters in stage 1, while the number of clusters in stage 2 is chosen using Eq 9. We use the F-measure evaluation to visualise this final result. PSC is again provided as a benchmark with *K* = 1427.

**Fig 11:**
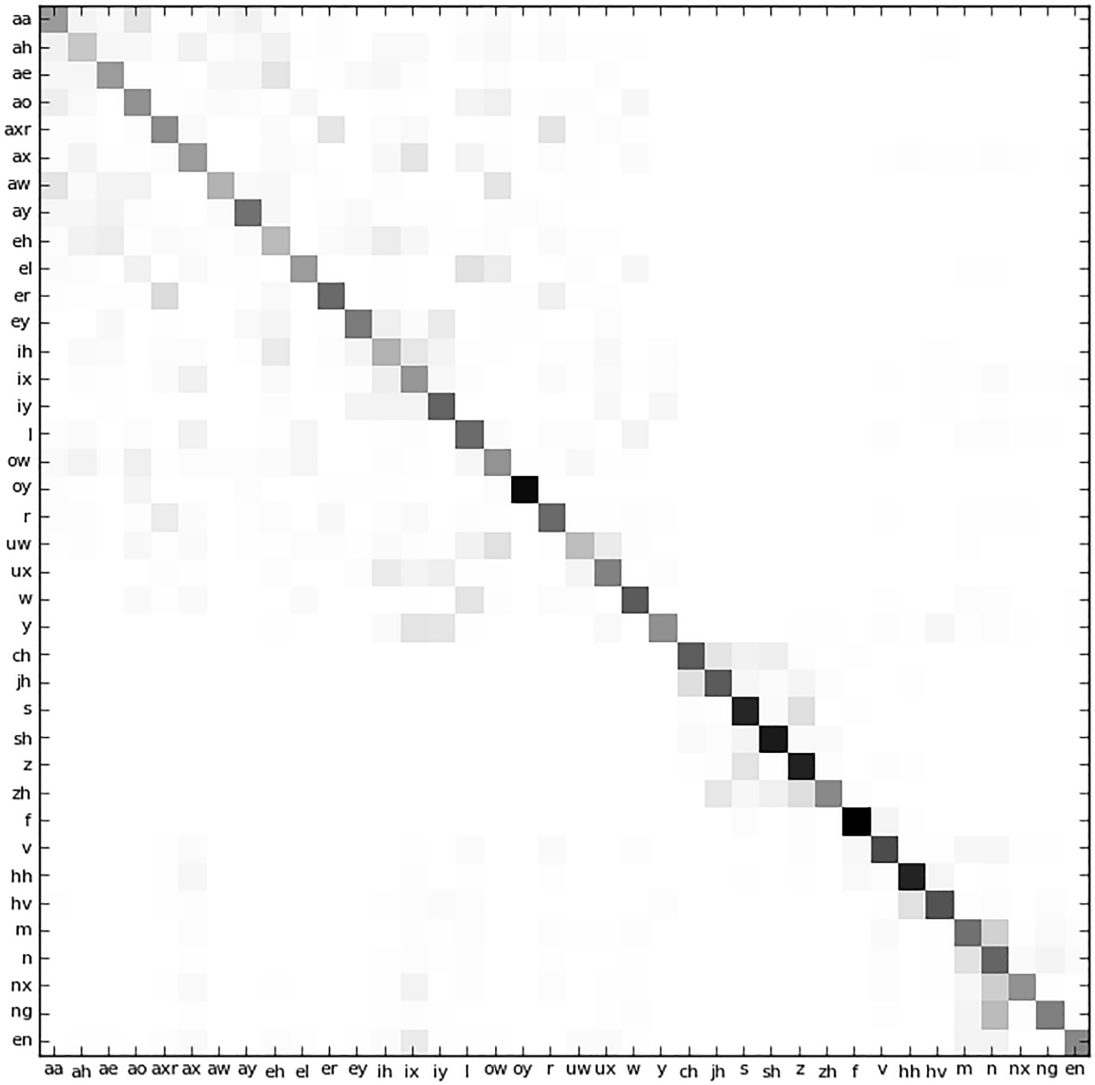
Confusion matrix of base phones of the large TIMIT dataset.

Since a comparison of the results in Table 7 with the AHC baseline is not feasible, we make use of a confusion matrix to visualise the similarity of the clustered acoustic segments. Fig 11 shows how often the derived clusters coincide with the known phone labels.

**Table 7:** Performance of the proposed method on the Large Set.

**No. of iterations**	**F-measure**	**No. of clusters**
1	0.007113	1264
2	0.01508	1450
3	0.01663	1427
4	0.01713	1395
5	0.01822	1423

To plot this figure, we considered just the centre phone of the triphone, that is, the triphone without its context. Clusters were associated with a phone when that phone was the dominant member of the cluster. Four phones *‘em’, ‘eng’, ‘h’* and *‘uh’* were not dominant in any of the 1423 clusters. For this reason the matrix has only 38 dimensions. We further show in the following section that PSC performance on Large Set at *K* = 1427 or at the third iteration is 0.01039.

Fig 11 clearly shows a dominant diagonal, indicating good correspondence between the clusters and the known phonetic labels. This is an indication that the clustering successfully produced groups of audio segments that show a high correspondence with the ground truth phonetic labels. Where phones are confused, they are usually between similar sounds such as *‘n’* and *‘ng’* or between *‘m’* and *‘n’*.

### Comparison with parallel spectral clustering

To evaluate the success of MAHC, we provide corresponding benchmark performance by the parallel spectral clustering method reported by Chen *et al* [18]. Since spectral clustering commonly requires the number of clusters *K* to be known in advance, we use the values of *K* determined by the MAHC algorithm.

Since we have found that MAHC approximates classical AHC performance after the third iteration, we benchmark the PSC approach in Table 8 using values of *K* from the third iteration.

**Table 8:** F-Measure performances of the L method based MAHC and the PSC algorithm.

Dataset	No. of Clusters	MAHC: F-Measure	PSC: F-Measure
Small Set A	208	0.1109	0.1210
Small Set B	305	0.06344	0.05504
Medium Set	660	0.04761	0.02966
Large Set	1427	0.01663	0.01039

The use of the L method together with the empirically observed relationship expressed by Eq 9 allows a completely unsupervised application of MAHC for large datasets. In Table 8 we therefore use values of *K* which correspond to the number of clusters after the third iteration when using the L method in stage 1 and Eq 9 in stage 2. From these benchmark results we observe that, for Small Set A, PSC delivers better performance than MAHC. For Small Set B, the Medium Set and the Large Set, the MAHC reflects better performance. It should be borne in mind that, for a particular dataset, spectral clustering requires the correct user-determined value of *K* and the optimal empirically obtained value of *t*.

### Computational efficiency

**Fig 12:**
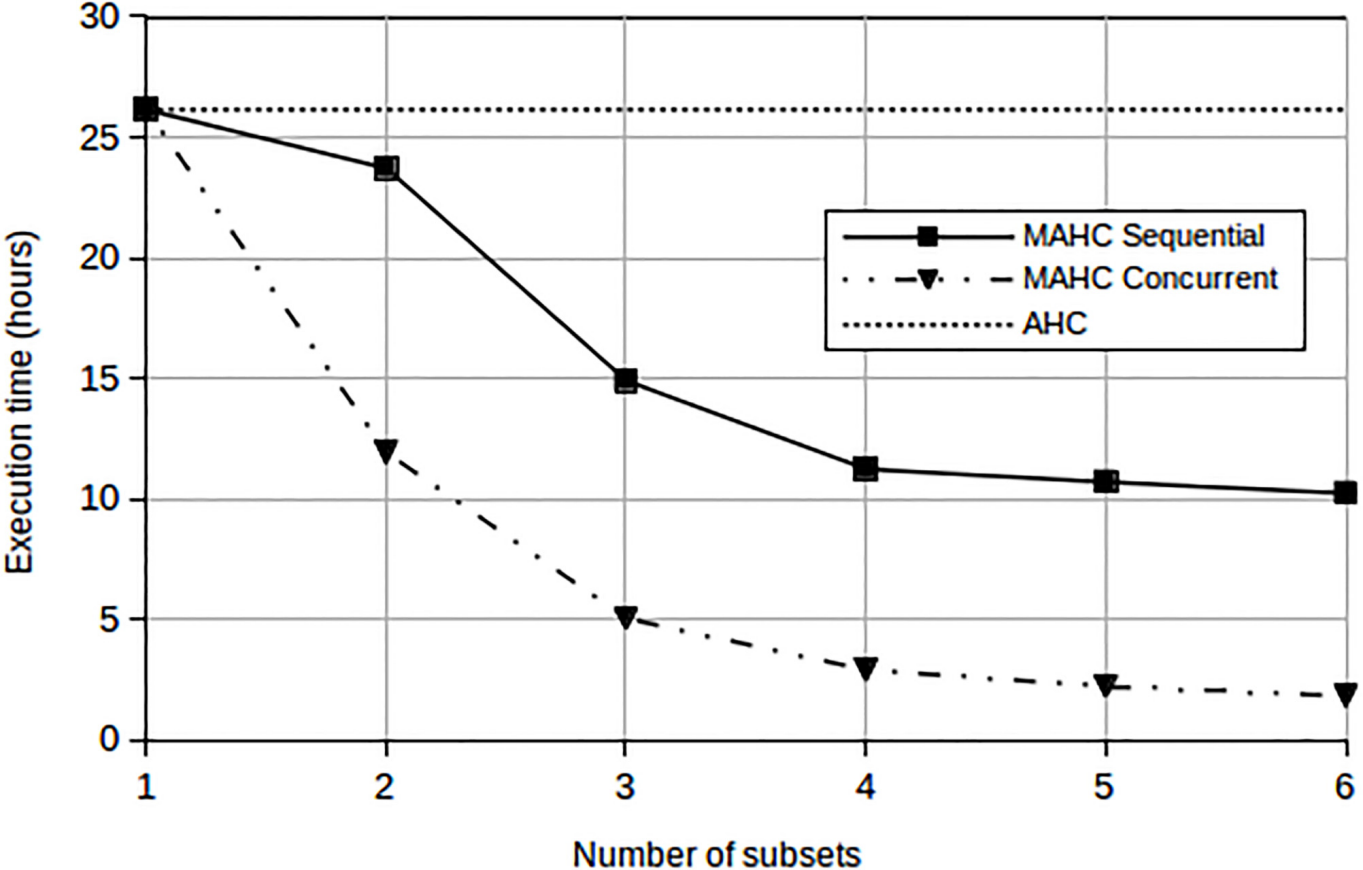
Influence of the number of subsets used by MAHC on the execution time. Classical AHC is included as a baseline.

This paper focuses on the reduction of the storage complexity of AHC in order to make its application to large datasets feasible, and on the performance implications of the proposed approximations. However, we have performed a small test using Small Set B to give an indication of the impact of the proposed method on execution time. We measured the execution time of the classical AHC process, which entails the generation of a full triangular similarity matrix, the Ward linkage computation, the creation of a dendrogram data structure and the L method computation for determining the cutoff (or the number of clusters). We also measured the execution time of one iteration of the MAHC algorithm, both when executed on a single processor and when executed concurrently on *P* processors, where *P* is the number of subsets used in stage 1 of the algorithm. In the former case, each of the *P* clustering steps constituting stage 1 of the algorithm are executed sequentially. The results are shown in Fig 12.

From Fig 12 we observe that, the execution time of each iteration of the MAHC algorithm is less than that of the classical AHC even when run on a single processor. By taking advantage of parallel computation, the execution time is further reduced to just 2 hours when data is split into 6 subsets. We observe the indication that the MAHC computational complexity at *P* × *O*(*N* ^2^/*P* ^2^) practically leads to a reduction in the execution time when compared with the classical AHC algorithm.

## Conclusions

We have proposed a multi-level agglomerative hierarchical clustering (MAHC) algorithm that is better suited to large datasets than classical agglomerative hierarchical clustering (AHC). The algorithm is based on a split of the dataset into a number of subsets that are clustered separately using AHC. Subsequently, the results of these separate clustering operations are merged and then used to obtain a new split of the dataset into independent subsets. Experiments show that the iteration of these steps leads to a convergence in the clusters and clustering performance. When using speech segments from the TIMIT corpus, experiments show that the performance of MAHC matches and often surpasses that of AHC. Furthermore MAHC was also demonstrated to offer some improvement on parallel spectral clustering under matching experimental conditions, and that this improvement was greatest for the largest dataset. We therefore conclude that MAHC is a promising candidate when clustering large datasets.

## References

[1] Jain AK. Data Clustering: 50 years beyond K-means. Pattern Recognition Letters. 2010;31(8):651–666. 10.1016/j.patrec.2009.09.011

[2] Jain AK, Dubes RC. Algorithms for Clustering Data. Upper Saddle River, NJ, USA: Prentice-Hall, Inc.; 1988.

[3] Manning CD, Raghavan P. Introduction to Information Retrieval. New York, USA: Cambridge University Press; 2008.

[4] Fung G. A Comprehensive Overview of Basic Clustering Algorithms; 2001.

[5] Jain AK, Murty MN, Flynn PJ. Data Clustering: A Review. ACM Computing Surveys. 1999;31(3):264–323. 10.1145/331499.331504

[6] Murtagh F, Contreras P. Methods of hierarchical clustering. arXiv preprint arXiv:11050121 2011;.

[7] Xu R, Wunsch D. Survey of clustering algorithms. Neural Networks, IEEE Transactions on. 2005;16(3):645–678. 10.1109/TNN.2005.845141 15940994

[8] Fraley C, Raftery AE. How Many Clusters? Which Clustering Method? Answers Via Model-Based Cluster Analysis. The Computer Journal. 1998;41:578–588. 10.1093/comjnl/41.8.578

[9] Varshavsky R, Horn D, Linial M. Global considerations in hierarchical clustering reveal meaningful patterns in data. PloS one. 2008;3(5):e2247 10.1371/journal.pone.0002247 18493326 PMC2375056

[10] Yin J, Yang J, Guo Q. Evaluating the Feasibility of an Agglomerative Hierarchy Clustering Algorithm for the Automatic Detection of the Arterial Input Function Using DSC-MRI. PloS one. 2014;9(6):e100308 10.1371/journal.pone.0100308 24932638 PMC4059756

[11] Loewenstein Y, Portugaly E, Fromer M, Linial M. Efficient algorithms for accurate hierarchical clustering of huge datasets: tackling the entire protein space In: ISMB; 2008 p. 41–49. 10.1093/bioinformatics/btn174 PMC2718652 18586742

[12] Imperl B, Kacic Z, Horvat B, Zgank A. Clustering of Triphones using Phoneme Similarity Estimation for the Definition of a Multilingual Set of Triphones. Speech Communication. 2003;39(4):353–366. 10.1016/S0167-6393(02)00048-1

[13] Svendsen T, Soong FK. On the Automatic Segmentation of Speech Signals. In: Proc. ICASSP; 1987. p. 87–80.

[14] Paliwal KK. Lexicon-Building Methods for an Acoustic Sub-Word Based Speech Recognizer. In: Proc. ICASSP; 1990. p. 108–111.

[15] Singh R, Raj B, Stern RM. Automatic Generation of Subword Units for Speech Recognition Systems. IEEE Transactions on Speech and Audio Processing. 2002;10(2):89–99. 10.1109/89.985546

[16] Wang H, Lee T, Leung C, Ma B, Li H. Unsupervised Mining of Acoustic Subword Units With Segment-level Gaussian Posteriograms. In: Proc. of Interspeech; 2013. p. 2297–2301.

[17] Davel MH, Heerden CV, Kleyhans N, Barnard E. Efficient harvesting of Internet Audio for resource-scarce ASR. In: Proc. Interspeech. Florence, Italy; 2011.

[18] Chen WY, Song Y, Bai H, Lin CJ, Chang EY. Parallel Spectral Clustering in Distributed Systems. IEEE Transactions on Pattern Analysis and Machine Intelligence. 2011;33(3):568–586. 10.1109/TPAMI.2010.88 20421667

[19] Narasimha Murty M, Krishna G. A computationally efficient technique for data-clustering. Pattern Recognition. 1980;12(3):153–158. 10.1016/0031-3203(80)90039-4

[20] Suresh Babu VSS. Optimal number of levels for a multilevel clustering method. Pattern Recognition Letters. 1990;11(9):595–599. 10.1016/0167-8655(90)90011-P

[21] Tang CH, Huang AC, Tsai MF, Wang WJ. An efficient distributed hierarchical-clustering algorithm for large scale data. In: IEEE Computer Symposium (ICS), 2010 International; 2010. p. 869–874.

[22] Cobo G, García-Solórzano D, Morán JA, Santamaría E, Monzo C, Melenchón J. Using Agglomerative Hierarchical Clustering to Model Learner Participation Profiles in Online Discussion Forums. In: Proc. ACM 2Nd International Conference on Learning Analytics and Knowledge. Vancouver, British Columbia, Canada; 2012. p. 248–251.

[23] Soltanolkotabi M, Candes EJ. A geometric analysis of subspace clustering with outliers. The Annals of Statistics. 2012;40(4):2195–2238. 10.1214/12-AOS1034

[24] Cobo Rodríguez G. Parameter-free agglomerative hierarchical clustering to model learners’ activity in online discussion forums. Universitat Oberta de Catalunya. Internet Interdisciplinary Institute (IN3); 2014.

[25] Lee CH, Soong FK, Juang BH. A Segment Model Based Approach to Speech Recognition. In: Proc. ICASSP; 1988. p. 501–504.

[26] Shi J, Malik J. Normalized Cuts and Image Segmentation. IEEE Transactions on Pattern Analysis and Machine Intelligence. 1997;22:888–905.

[27] Amigo E, Gonzalo J, Artiles J, Verdejo F. A Comparison of Extrinsic Clustering Evaluation Metrics Based on Formal Constraints. Information Retrieval. 2009;12(4):461–486. 10.1007/s10791-008-9066-8

[28] Von Luxburg U. A tutorial on spectral clustering. Statistics and computing. 2007;17(4):395–416. 10.1007/s11222-007-9033-z

[29] Chen X, Cai D. Large Scale Spectral Clustering with Landmark-Based Representation In: AAAI; 2011. 10.1109/TCYB.2014.2358564 25265642

[30] Zhang T, Ramakrishnan R, Livny M. BIRCH: an efficient data clustering method for very large databases In: ACM SIGMOD Record. vol. 25; 1996 p. 103–114.

[31] Ward J Joe H. Hierarchical Grouping to Optimize an Objective Function. Journal of the American Statistical Association. 1963;58(301):236–244. 10.1080/01621459.1963.10500845

[32] Murtagh F, Legendre P. Ward’s Hierarchical Agglomerative Clustering Method: Which Algorithms Implement Ward’s Criterion? Journal of Classification. 2014;31(3):274–295.

[33] Day WE, Edelsbrunner H. Efficient algorithms for agglomerative hierarchical clustering methods. Journal of Classification. 1984;1(1):7–24. 10.1007/BF01890115

[34] Liu Y, Li Z, Xiong H, Gao X, Wu J, Wu S. Understanding and Enhancement of Internal Clustering Validation Measures. Cybernetics, IEEE Transactions on. 2013;43(3):982–994. 10.1109/TSMCB.2012.2220543 23193245

[35] Salvador S, Chan P. Determining the Number of Clusters/Segments in Hierarchical Clustering/Segmentation Algorithms. In: Proceedings of the 16th IEEE International Conference on Tools with Artificial Intelligence. ICTAI’04. IEEE Computer Society; 2004. p. 576–584.

[36] Larsen B, Aone C. Fast and Effective Text Mining Using Linear-time Document Clustering. In: Proc. of the fifth ACM SIGKDD. New York, USA; 1999. p. 16–22.

[37] Halberstadt AK, Glass JR. Heterogeneous acoustic Measurements for Phonetic Classification. In: Proc. of Eurospeech 97; 1997.

[38] Myers C, Rabiner LR, Rosenberg AE. Performance Tradeoffs in Dynamic Time Warping Algorithms for Isolated Word Recognition. IEEE Transactions on Acoustics, Speech, and Signal Processing. 1980;28(6):623–635. 10.1109/TASSP.1980.1163491

[39] Yu F, Dong K, Chen F, Jiang Y, Zeng W. Clustering Time Series with Granular Dynamic Time Warping Method. In: Proceedings of the 2007 IEEE International Conference on Granular Computing. GRC’07. Washington, DC, USA: IEEE Computer Society; 2007. p. 393–398.

[40] Amigo E, Gonzalo JA J, Verdejo F. A Comparison of Extrinsic Clustering Evaluation Metrics Based on Formal Constraints. Information Retrieval. 2009;12(4):461–486. 10.1007/s10791-008-9066-8

[41] Wu J, Xiong H, Chen J. Towards Understanding Hierarchical Clustering: A Data Distribution Perspective. Neurocomputing. 2009 6;72(10–12):2319–2330. 10.1016/j.neucom.2008.12.011

[42] Bombrun L, Vasile G, Gay M, Totir F. Hierarchical segmentation of polarimetric SAR images using heterogeneous clutter models. Geoscience and Remote Sensing, IEEE Transactions on. 2011;49(2):726–737. 10.1109/TGRS.2010.2060730

[43] Lopes C, Perdigao F. Phone Recognition on the TIMIT Database; 2011. Available from: http://www.intechopen.com/books/howtoreference/speech-technologies/phoneme-recognition-on-the-timit-database.

[44] Young SJ, Young S. The HTK hidden Markov model toolkit: Design and philosophy. Citeseer; 1993.

